# Comparison of pre-workout nitric oxide stimulating dietary supplements on skeletal muscle oxygen saturation, blood nitrate/nitrite, lipid peroxidation, and upper body exercise performance in resistance trained men

**DOI:** 10.1186/1550-2783-7-16

**Published:** 2010-05-06

**Authors:** Richard J Bloomer, Tyler M Farney, John F Trepanowski, Cameron G McCarthy, Robert E Canale, Brian K Schilling

**Affiliations:** 1 Cardiorespiratory/Metabolic Laboratory, Department of Health and Sport Sciences, The University of Memphis, Memphis, TN 38152, USA; 2 Exercise Neuromechanics Laboratory, Department of Health and Sport Sciences, The University of Memphis, Memphis, TN 38152, USA

## Abstract

**Background:**

We compared Glycine Propionyl-L-Carnitine (GlycoCarn^®^) and three different pre-workout nutritional supplements on measures of skeletal muscle oxygen saturation (StO_2_), blood nitrate/nitrite (NOx), lactate (HLa), malondialdehyde (MDA), and exercise performance in men.

**Methods:**

Using a randomized, double-blind, cross-over design, 19 resistance trained men performed tests of muscular power (bench press throws) and endurance (10 sets of bench press to muscular failure). A placebo, GlycoCarn^®^, or one of three dietary supplements (SUPP1, SUPP2, SUPP3) was consumed prior to exercise, with one week separating conditions. Blood was collected before receiving the condition and immediately after exercise. StO_2 _was measured during the endurance test using Near Infrared Spectroscopy. Heart rate (HR) and rating of perceived exertion (RPE) were determined at the end of each set.

**Results:**

A condition effect was noted for StO_2 _at the start of exercise (p = 0.02), with GlycoCarn^® ^higher than SUPP2. A condition effect was also noted for StO_2 _at the end of exercise (p = 0.003), with SUPP1 lower than all other conditions. No statistically significant interaction, condition, or time effects were noted for NOx or MDA (p > 0.05); however, MDA decreased 13.7% with GlycoCarn^® ^and increased in all other conditions. Only a time effect was noted for HLa (p < 0.0001), with values increasing from pre- to post-exercise. No effects were noted for HR, RPE, or for any exercise performance variables (p > 0.05); however, GlycoCarn^® ^resulted in a statistically insignificant greater total volume load compared to the placebo (3.3%), SUPP1 (4.2%), SUPP2 (2.5%), and SUPP3 (4.6%).

**Conclusion:**

None of the products tested resulted in favorable changes in our chosen outcome measures, with the exception of GlycoCarn^® ^in terms of higher StO_2 _at the start of exercise. GlycoCarn^® ^resulted in a 13.7% decrease in MDA from pre- to post-exercise and yielded a non-significant but greater total volume load compared to all other conditions. These data indicate that 1) a single ingredient (GlycoCarn^®^) can provide similar practical benefit than finished products containing multiple ingredients, and 2) while we do not have data in relation to post-exercise recovery parameters, the tested products are ineffective in terms of increasing blood flow and improving acute upper body exercise performance.

## Background

The use of nutritional supplements for sport continues to increase [1], with athletes and recreationally active trainees routinely seeking methods to improve performance. In particular, the category of sport supplements known as the "pre-workout" class appears to be a staple in the regimen of many athletes, bodybuilders and strength athletes in particular. These products typically contain a combination of several (30+) ingredients, and usually contain stimulants (e.g., caffeine), energy-producing agents (e.g., creatine), agents that act as hydrogen ion buffers (e.g., beta alanine), protein recovery nutrients (e.g., amino acids), antioxidants, and nitric oxide precursors (e.g., arginine). In relation to the latter, an entire class of sport supplement ("nitric oxide boosters") has been built around the theoretical increase in nitric oxide following intake of L-arginine, and the supposed but unsubstantiated correlation between increased circulating nitric oxide and improved exercise performance and recovery [2]. Companies developing and selling such products boldly claim that a *single use *of the product will rapidly and dramatically increase circulating nitric oxide and result in an improvement in blood flow, muscle "pump", and exercise performance.

Collectively, hundreds of studies have been conducted testing the commonly used pre-workout ingredients *in isolation*, many with reported positive findings related to the chosen outcome measures. For example, caffeine intake prior to exercise has been reported to improve both aerobic and anaerobic exercise performance, although results are mixed [3,4]. The dosage used in most studies has ranged from 3-7mg∙kg^-1 ^consumed prior to exercise [3,4], although higher amounts have certainly been used in many studies. Creatine is another well-studied nutrient noted to improve high intensity exercise performance [5]. The traditional dosage used in most studies is 5 grams per day, usually taken for a series of days/weeks leading up to the exercise test protocol. One relatively new ingredient which shows promise is beta alanine. This agent has been reported in most [6-9], but not all studies [10,11], to decrease lactate accumulation and/or aid in exercise performance. The dosage used in most studies ranges from 3-6 grams per day, usually taken for a series of days/weeks leading up to the exercise test protocol. The novel ingredient Glycine Propionyl-L-Carnitine (GlycoCarn^®^) has been reported recently to improve repeated sprint cycle performance and reduce the blood lactate response to exercise when consumed in a single dosage of 4.5 grams [12]. We have also reported an increase in nitric oxide (measured as nitrate/nitrite) when subjects received GlycoCarn^® ^at a daily dosage of 4.5 grams for either four [13] or eight [14] weeks. Lastly, several antioxidant agents have been reported to decrease the oxidative stress response to exercise [15], and are believed to promote exercise recovery; hence, these are often included within some pre-workout supplements.

While the data obtained from investigations focused on the study of individual ingredients indeed support the use of such ingredients when included at the correct dosages, most finished products contain a combination of multiple ingredients at extremely low dosages. Moreover, most of the current pre-workout dietary supplements claim to increase nitric oxide production, which in turn will increase blood flow, muscle pumps, and overall exercise performance.

Two concerns arise when considering the above claims: 1) Aside from GlycoCarn^® ^when used at a daily dosage of 4.5 grams, there are no peer reviewed and published data in scientific manuscript format pertaining to a dietary supplement, consumed in oral form by healthy subjects, to support an increase in nitric oxide; 2) Even if data were available demonstrating an increase in blood nitric oxide following dietary supplement intake, no evidence exists to support the claim that increased circulating nitric oxide leads to better muscle pumps or improved exercise performance. Such a claim is premature and requires laboratory testing in order to be substantiated. Therefore, the purpose of the present study was to compare GlycoCarn^® ^and three different popular pre-workout "nitric oxide stimulating" nutritional supplements on measures of skeletal muscle oxygen saturation (StO_2_), blood nitrate/nitrite (NOx), blood lactate (HLa), malondialdehyde (MDA), and exercise performance in a sample of resistance trained men. It should be understood that no attempt was made to determine the effects of the tested products on post-exercise recovery components. Therefore, no conclusions should be made with regards to these variables.

## Methods

### Subjects

Nineteen resistance trained men were recruited from the University of Memphis and local surrounding community and completed all aspects of this study. All men performed resistance exercise a minimum of three days per week for the past 12 months, with the majority of subjects training more frequently and for much longer than the past 12 months (Table 1). Subjects were not current smokers, and did not have cardiovascular, metabolic, or orthopedic problems that might affect their ability to perform submaximal and maximal resistance exercise. Subject characteristics are presented in Table 1. Health history, drug and dietary supplement usage, and physical activity questionnaires were completed by all subjects to determine eligibility. Subjects were instructed to maintain their current training and nutritional regimen throughout the course of the study period, with the exception of the 48 hours prior to each test session in which they were instructed not to perform any strenuous exercise. The study was approved by the university committee for human subject research and all subjects provided both verbal and written consent.

**Table 1:** Descriptive characteristics of 19 resistance trained men.

Variable	Value
Age (yrs)	24 ± 4
Height (cm)	176 ± 5
Weight (kg)	80 ± 7
Body mass index (kg∙m^-2^)	26 ± 3
Body fat (%)*	13 ± 3
Waist:Hip	0.86 ± 0.04
Years resistance exercise	7 ± 4
Hours/wk resistance exercise	4 ± 2
Bench press 1-RM (kg)	150 ± 39
Resting heart rate (bpm)	65 ± 13
Resting systolic blood pressure (mmHg)	119 ± 11
Resting diastolic blood pressure (mmHg)	69 ± 8

Data are mean ± SD.

*Determined from 7-site skinfold analysis use Lange calipers and Siri equation

### Design

This study involved a randomized, placebo controlled, cross-over, double blind design. During the first visit to the laboratory, subjects gave written informed consent and completed health and physical activity questionnaires. Additionally, the subjects' height, weight, and body composition (via 7 site skinfold test) was measured. Heart rate and blood pressure were recorded following a 10 minute period of quiet rest. Familiarization trials were performed for the bench press throw (using a ProSpot^® ^device; ProSpot Fitness, Norcross, GA). A maximal test in the bench press exercise was performed using a supine Hammer Strength™ bench press apparatus, in order to determine subjects' one repetition maximum (1RM). Guidelines from the National Strength and Conditioning Association were followed [16]. Testing began, as described below, within one week after the completion of this screening visit.

### Conditions

**Figure 1:**
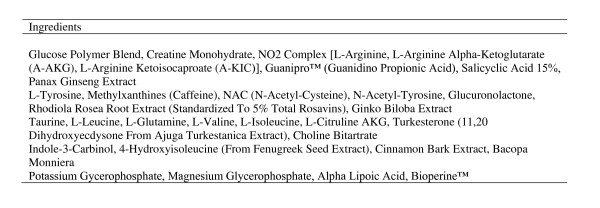
**Supplement 1 ingredients (per one serving)**.

**Figure 2:**
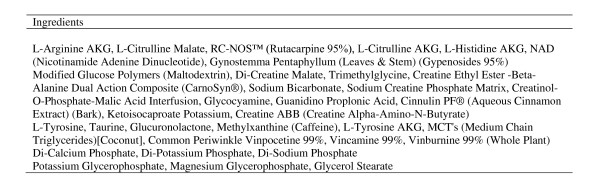
**Supplement 2 ingredients (per one serving)**.

**Figure 3:**
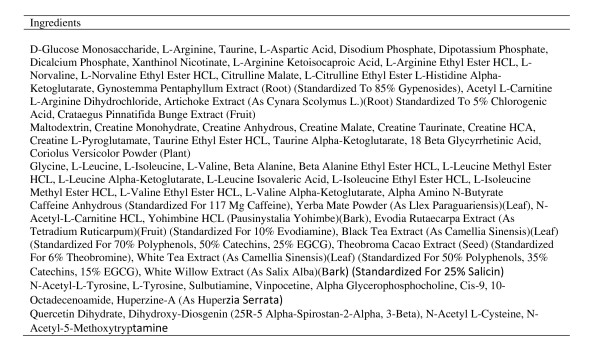
**Supplement 3 ingredients (per one serving)**.

Subjects underwent the exact exercise testing protocol a total of six times, each visit separated by one week. The conditions included a placebo powder (16 grams of maltodextrin), Glycine Propionyl-L-Carnitine (16 grams of maltodextrin + 4.5 grams of GlycoCarn^®^; Sigma-tau HealthScience, Gaithersburg, MD), Supplement 1 (SUPP1--lot # 9084; expiration 04/2012; see Figure 1), Supplement 2 (SUPP2--lot #62149A; expiration 06/2011; see Figure 2), and Supplement 3 (SUPP3--lot # 907495; expiration 09/2011; see Figure 3). Subjects were simply told that they were receiving a "pre-workout" supplement. For each of the supplements used for comparison, two servings were provided to subjects. Sixteen grams of maltodextrin was added to the GlycoCarn^® ^and also used as the placebo in an attempt to match the mean amount of maltodextrin contained within the supplements used in comparison (when considering our two-serving dosage). All conditions were mixed into 12 ounces of water and consumed 30 minutes prior to the start of the exercise test protocol, with the exception of the GlycoCarn^® ^condition which was consumed 60 minutes prior to the start of exercise. The time of administration of each condition was similar to the recommended time of intake provided on the product label, while a recent study using GlycoCarn^® ^for performance improvement had subjects consume this condition 90 minutes prior to exercise [12]. Our rationale for the change to 60 minutes prior to exercise was based on our inclusion of maltodextrin to the GlycoCarn^® ^in the current design and the fact that the added carbohydrate may have enhanced uptake of the GlycoCarn^®^, as well as the fact that we wanted to maintain as much similarity in the treatment protocol as possible. Prior to using any of the above five conditions, all subjects underwent an identical test protocol using water only. This was to serve as a baseline familiarization trial to the protocol, as we have previously noted that even in well trained men, such a protocol as used in the present design requires one session in order to fully familiarize subjects to the exercise movements and the volume of exercise (unpublished findings). Hence, a total of six sessions of the exercise protocol were performed by all subjects. It should be noted that the baseline condition, although presented within the results section for comparison purposes, was not used in the statistical analysis.

All conditions were provided in powder form and were fruit punch flavor. The placebo and GlycoCarn^® ^conditions were produced and then packaged into individual servings by Tishcon Corporation (Westbury, NY). The three supplements used for comparison were purchased from a local General Nutrition Center store in containers. To ensure precision of dosing, each of these three conditions was weighed on a laboratory grade balance prior to mixing in water. Again, two servings of each condition were used in this design. Our rationale for this was based on the fact that the majority of users of such supplements use 2-3 servings rather than one. In fact, the label instructions for use of these products indicate a serving size between 1 and 3 servings. Unlike GlycoCarn^®^, which is obviously a single ingredient (mixed with maltodextrin in the present design), the supplements contained numerous ingredients (as can be seen in Figures 1, 2, and 3), some of which are stimulants.

### Exercise Test Protocol

For all six test days, subjects reported to the lab following a minimum of an eight hour overnight fast. After arrival to the lab, a blood sample was obtained following a 10 minute period of rest. Subjects then rated their perceived and subjective level of muscle "pump" in the upper body using a visual analog scale (0 = no pump; 10 = the most intense pump ever experienced). This subjective measure was explained to subjects as an intense feeling of swell within the muscle, in such a way that the muscle feels bigger, fuller, and harder. As all subjects were resistance trained men, all had a full understanding of the described feeling. A circumference measure of the upper torso was also taken twice using a tension regulated tape measure (across the nipple line with the shirt removed), and the mean of two measures was recorded. Subjects stood relaxed during these measures with their arms at their sides. These exact measures for muscle pump and circumference were taken a second time, within five minutes of completing the exercise protocol. Subjects then consumed their assigned condition and prepared for the performance tests. During this time, subjects were fitted with a heart rate monitor to be worn during the entire exercise test protocol. Following the required time (60 minutes for GlycoCarn^® ^and 30 minutes for all other conditions), the performance tests were performed in the order described below. No other food or calorie-containing drinks were allowed during testing, but water was allowed ad libitum for the first session and matched for all conditions and days of testing. Although water intake was matched for each subject for each condition, we did not measure hydration status of subjects. This may be considered a limitation of the present work, as hydration status has been reported to influence the hormonal environment associated with acute resistance exercise [17], which could have possibly influenced our outcome measures.

### Performance Testing

As a measure of muscular power, bench press throws were performed using the ProSpot^® ^device. Following a warm-up of 10% of their predetermined 1RM, subjects performed three throws using 30% of 1RM. Ninety seconds of rest was provided between each throw. The best attempt of the three throws was recorded and used in the data analysis. A detailed description of this assessment is provided elsewhere [18]; however basic procedures were as follows. Kinetic and kinematic data were acquired through the combination of a modified floor scale (Roughdeck, Rice Lake Weighing Systems, Rice Lake, WI) and a linear velocity/position transducer (VP510, Unimeasure, Corvallis, OR). The linear transducer was mounted superior to the barbell and was centrally tethered to the barbell. Measurements of force and velocity were measured directly by the modified floor scale and linear transducer, respectively. Power was calculated indirectly via inverse dynamic equations within our acquisition software (DataPac 5).

Following the bench press throws, a sensor was placed on subjects' dominant arm anterior deltoid muscle for a measure of muscle tissue oxygen saturation using Near Infrared Spectroscopy (NIRS), as described below. Subjects then performed the bench press test which involved 10 sets in the Hammer Strength™ supine bench press exercise using a load equal to 50% of 1RM. This same intensity of resistance exercise has been used in many previous studies, and also used in one of the few studies incorporating the measure of NIRS [19]. All sets of exercise were performed to a point of momentary muscular failure, with 120 seconds of rest between each set. Total repetitions performed for each set were recorded, and total and mean volume load (reps × load) was calculated. Immediately at the conclusion of each set, heart rate and perceived exertion (using the 6-20 Borg scale) were recorded. The mean values over all 10 sets for heart rate and perceived exertion for each test day were computed and used in data analysis.

### Near Infrared Spectroscopy (NIRS)

Muscle tissue oxygen saturation was measured continuously during the bench press protocol (both work and rest) using the InSpectra™ Tissue Oxygenation Monitor (Hutchinson Technology; Hutchinson, MN). This system uses near infrared spectroscopy (NIRS; i.e., calibrated wavelengths of near infrared light) to noninvasively illuminate the tissue below a sensor that is placed on the skin surface. This device provides quantification of the ratio of oxygenated hemoglobin to total hemoglobin in the microcirculation of the volume of illuminated tissue. The system does this via use of a sensor attached to the subjects' skin (anterior deltoid in the present design). Through pilot testing it was determined that the system was most sensitive when the sensor was applied to the anterior deltoid muscle (as opposed to the pectoralis major or pectoralis minor muscle).

NIRS is widely used around the world for monitoring tissue oxygen saturation in trauma and critical care medicine; however, it has only been used in a few exercise related studies [19-21], and may have some limitations compared to a more sophisticated tool such as magnetic resonance imaging [22]. Moreover, it should be understood that this device is not directly measuring blood flow in the same manner as using flow mediated dilation via ultrasound technology. Our rationale for using this instrument in the present design was that if the conditions actually promoted an increase in blood flow (via any mechanism), then the amount of oxygen saturation at the start of each set of exercise may be greater and the percent of desaturation may be less at the conclusion of each set of exercise. Based on this rationale, we recorded the precise starting oxygen saturation (StO_2 _start) and ending oxygen saturation (StO_2 _end) for each of the 10 sets of exercise. The difference was also calculated for each set.

It has been suggested that carnitine supplementation may improve blood flow regulation and the delivery of oxygen to muscle tissue during and after exercise [23]. Such an increase in oxygen delivery may decrease the degree of tissue ischemia and subsequent free radical formation, leading to less oxidation of cellular lipids and other macromolecules [24]. Likewise, although not yet supported by the scientific literature, it is suggested in lay press publications that an increase in oxygen delivery to muscle tissue during exercise will improve physical performance. The collective measures employed in the present study address these issues.

### Blood Collection and Biochemistry

At two times (pre-intake of condition and fasting; within one minute following the completion of set 10 of bench press exercise) blood was collected (~7mL) from subjects' antecubital veins using a needle and collection tube. Single samples were immediately analyzed for whole-blood lactate using an Accutrend portable lactate analyzer (Roche Diagnostics, Mannheim, Germany). The remainder of whole blood was immediately processed for plasma and stored at -70°C until analysis within three months of collection. The following assays for nitrate/nitrite and malondialdehyde were performed in duplicate.

Nitrate/nitrite was analyzed in plasma using a commercially available colorimetric assay kit (Catalog#: 780001; Caymen Chemical, Ann Arbor, MI), according to the procedures provided by the manufacturer. After being thawed, plasma samples were centrifuged at 10,000xg for 5 minutes in a refrigerated centrifuge (4°C). Following the addition of a nitrate reductase co-factor to each diluted sample, nitrate reductase was added and the mixture was incubated for three hours to allow for the full conversion of nitrate to nitrite. Greiss reagent was then added, which converts nitrite into a deep purple azo compound. The absorbance was then detected photometrically at 540nm. Quantification was performed with a calibration curve. The coefficient of variation for this assay in our laboratory is <8%. The detection limit, as per the manufacturer, is ≥2.5 μM.

Malondialdehyde was analyzed in plasma following the procedures of Jentzsch et al. [25] using reagents purchased from Northwest Life Science Specialties (Vancouver, WA). Specifically, 75 μL of plasma was added to microcentrifuge reaction tubes with the addition of 3 μL of butylated hydroxytoluene in methanol to minimize *ex vivo *lipid peroxidation. 75 μL of 1M phosphoric acid and 75 μL of 2-thiobarbituric acid reagent was added to each reaction tube and mixed thoroughly. Samples and reagents were incubated for 60 minutes at 60°C. Following incubation, tubes were removed and the reaction mixture was transferred to a microplate and the absorbance read using a spectrophotometer at both 535 and 572nm to correct for baseline absorption. Malondialdehyde equivalents were calculated using the difference in absorption at the two wavelengths. Quantification was performed with a calibration curve using tetramethoxypropane in a stabilizing buffer. The coefficient of variation for this assay in our laboratory is <6%. The detection limit, as per the manufacturer, is 0.1 μM.

### Physical Activity and Dietary Intake

Subjects were asked to refrain from strenuous physical activity during the 48 hours before test days. Subjects were asked to record all food and drink consumed during the 24 hours prior to each test day. Upon receipt of the first 24 hour diet record, subjects received a copy and were asked to duplicate this intake during the 24 hour period immediately prior to all subsequent test days. However, to verify that subjects consumed similar intakes, they recorded food and drink for the 24 hours prior to each test day and all records were analyzed for total calories, protein, carbohydrate, fat, vitamin C, vitamin E, and vitamin A (Food Processor SQL, version 9.9, ESHA Research, Salem, OR).

### Statistical Analysis

All performance data, mean HR, mean RPE, and dietary data were analyzed using an analysis of variance (ANOVA). Blood HLa, NOx, MDA, subjective muscle pump, and circumference data were analyzed using a 5 (condition) × 2 (time) ANOVA. The StO_2 _data (start, end, difference) were first analyzed using a 5 (condition) × 10 (set number) ANOVA. The data were then collapsed by set number and simply analyzed using an ANOVA in order to compare conditions without considering set number. Post hoc testing was performed using the procedures of Tukey. The outcome data are presented as mean ± standard error of the mean. Subject descriptive characteristics are presented as mean ± standard deviation. All analyses were performed using JMP statistical software (version 4.0.3, SAS Institute, Cary, NC). Statistical significance was set at P ≤ 0.05.

## Results

### Dietary Intake

Dietary data did not differ between conditions for total kilocalories (p = 0.83), protein (p = 0.99), carbohydrate (p = 0.84), fat (p = 0.43), vitamin C (p = 0.91), vitamin E (p = 0.58), or vitamin A (p = 0.41). Data are presented in Table 2.

**Table 2:** Dietary data of 19 resistance trained men receiving placebo or supplement in a cross-over design.

Variable	Baseline	Placebo	GlycoCarn^®^	SUPP1	SUPP2	SUPP3
Kilocalories	2352 ± 212	2592 ± 216	2881 ± 245	2617 ± 222	2915 ± 272	2795 ± 248
Protein (grams)	127 ± 19	140 ± 19	138 ± 18	134 ± 21	138 ± 18	137 ± 17
Carbohydrate (grams)	288 ± 31	295 ± 33	353 ± 38	335 ± 38	334 ± 37	320 ± 33
Fat (grams)	79 ± 9	98 ± 13	105 ± 13	86 ± 9	119 ± 14	107 ± 13
Vitamin C (mg)	102 ± 25	68 ± 16	88 ± 15	85 ± 30	68 ± 18	85 ± 17
Vitamin E (mg)	6 ± 2	5 ± 1	6 ± 1	7 ± 2	9 ± 2	7 ± 2
Vitamin A (RE)	516 ± 138	303 ± 76	584 ± 148	511 ± 130	371 ± 79	588 ± 174

Data are mean ± SEM.

No statistically significant difference noted between conditions for kilocalories (p = 0.83), protein (p = 0.99), carbohydrate (p = 0.84), fat (p = 0.43), vitamin C (p = 0.91), vitamin E (p = 0.58), or vitamin A (p = 0.41).

Values are for the 24 hour period immediately preceding each test condition.

### Performance Measures

No statistically significant differences were noted between conditions for bench press power (p = 0.93), reps performed during the first set (p = 0.99), total reps performed (p = 0.98), mean reps performed (p = 0.98), total volume load (p = 0.99), mean volume load (p = 0.99), mean heart rate over the 10 sets (p = 0.56), or mean perceived exertion over the 10 sets (p = 0.98). Although not statistically significant, the following are noted: SUPP1 resulted in the greatest power output during the bench press throws, with values higher than the placebo (5.8%), GlycoCarn^® ^(2.5%), SUPP2 (0.4%), and SUPP3 (1.5%). Likewise, GlycoCarn^® ^resulted in the greatest total volume load during the 10 set protocol, with values higher than the placebo (3.3%), SUPP1 (4.2%), SUPP2 (2.5%), and SUPP3 (4.6%). Mean HR was highest with SUPP2, with values higher than the placebo (8.4%), GlycoCarn^® ^(5.2%), SUPP1 (6.0%), and SUPP3 (3.6%). Other variables were essentially the same between conditions. Data are presented in Table 3.

**Table 3:** Exercise performance data of 19 resistance trained men receiving placebo or supplement in a cross-over design.

Variable	Baseline	Placebo	GlycoCarn^®^	SUPP1	SUPP2	SUPP3
Bench press power (W)	1029 ± 51	1019 ± 47	1052 ± 50	1078 ± 53	1073 ± 49	1062 ± 52
Reps 1^st ^set	25 ± 1	25 ± 1	26 ± 1	26 ± 1	26 ± 1	26 ± 1
Total reps	101 ± 6	105 ± 7	109 ± 6	104 ± 6	106 ± 5	104 ± 6
Mean reps	10.1 ± 0.6	10.5 ± 0.7	10.9 ± 0.6	10.4 ± 0.6	10.6 ± 0.5	10.4 ± 0.6
Total volume load (kg)	7221 ± 550	7495 ± 545	7746 ± 528	7432 ± 559	7558 ± 513	7407 ± 499
Mean volume load (kg)	722.1 ± 55.0	749.5 ± 54.5	774.6 ± 52.8	743.2 ± 55.9	755.8 ± 51.3	740.7 ± 49.9
Heart rate* (bpm)	131 ± 3	135 ± 4	134 ± 4	138 ± 3	142 ± 4	137 ± 4
Perceived exertion* (6-20)	14.7 ± 0.6	14.8 ± 0.4	14.7 ± 0.4	14.8 ± 0.4	14.6 ± 0.4	14.8 ± 0.4

Data are mean ± SEM.

No statistically significant difference noted between conditions for bench press power (p = 0.93), reps 1^st ^set (p = 0.99), total reps (p = 0.98), mean reps (p = 0.98), total volume load (p = 0.99), mean volume load (p = 0.99), heart rate (p = 0.56), or perceived exertion (p = 0.98).

*Heart rate and perceived exertion recorded at the end of each of the 10 sets of bench press exercise. Mean data presented in table.

### Muscle Tissue Oxygen Saturation

When considering the condition × set number ANOVA, the following was noted: For StO_2 _at the start of exercise, no condition × set number interaction was noted (p = 1.00). A condition effect was noted (p = 0.02), with GlycoCarn^® ^higher than SUPP2 (p < 0.05). A time effect was also noted (p < 0.0001), with set number one lower than all other sets (p < 0.05). For StO_2 _at the end of exercise, no condition × set number interaction was noted (p = 1.00). A condition effect was noted (p = 0.003), with SUPP1 lower than all other conditions (p < 0.05). A time effect was also noted (p = 0.002), with set number one lower than sets 5-10 (p < 0.05). For StO_2 _difference (start-end), no condition × set number interaction was noted (p = 1.00). A condition effect was noted (p = 0.004), with SUPP1 greater than all other conditions (p < 0.05). No time effect was noted (p = 0.94). Data are presented in Table 4.

**Table 4:** Muscle tissue oxygen saturation data for 10 sets of bench press exercise in 19 resistance trained men receiving placebo or supplement in a cross-over design.

Variable† *Condition*	Set 1**	Set 2	Set 3	Set 4	Set 5	Set 6	Set 7	Set 8	Set 9	Set 10
StO_2 _start (%) *Baseline*	85.2 ± 1.1	90.2 ± 1.3	91.0 ± 1.0	91.2 ± 1.1	91.4 ± 0.9	91.8 ± 0.9	91.9 ± 0.8	92.2 ± 0.8	92.1 ± 0.8	92.7 ± 0.7
StO_2 _end (%) *Baseline*	39.7 ± 3.5	44.8 ± 3.5	47.3 ± 4.2	47.3 ± 3.6	49.0 ± 3.0	49.7 ± 3.1	50.1 ± 2.7	47.8 ± 3.1	48.0 ± 2.8	48.0 ± 3.5
StO_2 _difference *Baseline*	45.5 ± 3.3	45.4 ± 3.4	43.7 ± 3.9	43.9 ± 3.5	42.4 ± 2.8	42.1 ± 2.8	41.8 ± 2.6	44.4 ± 2.9	44.1 ± 2.7	44.7 ± 3.3

StO_2 _start (%) *Placebo*	84.3 ± 1.3	91.0 ± 0.8	91.4 ± 0.8	91.8 ± 0.7	91.9 ± 0.8	92.3 ± 0.7	92.0 ± 0.7	92.2 ± 0.7	92.5 ± 0.6	92.5 ± 0.6
StO_2 _end (%) *Placebo*	39.2 ± 3.7	45.8 ± 4.2	48.8 ± 4.0	48.8 ± 4.5	50.1 ± 3.8	48.9 ± 4.3	49.0 ± 4.1	47.9 ± 4.1	50.1 ± 4.2	50.2 ± 4.0
StO_2 _difference *Placebo*	45.1 ± 3.5	45.2 ± 4.3	42.5 ± 4.2	43.0 ± 4.6	41.2 ± 3.8	43.3 ± 4.3	42.9 ± 4.1	44.3 ± 4.0	42.5 ± 4.1	42.3 ± 3.9

StO_2 _start (%) GlycoCarn^®^*	84.5 ± 0.8	92.1 ± 0.5	92.5 ± 0.5	92.5 ± 0.4	93.0 ± 0.4	92.9 ± 0.4	93.1 ± 0.5	92.9 ± 0.4	93.0 ± 0.4	92.7 ± 0.5
StO_2 _end (%) GlycoCarn^®^	40.5 ± 3.7	45.3 ± 3.6	46.9 ± 4.7	49.1 ± 3.9	49.9 ± 3.8	51.5 ± 3.7	50.5 ± 3.7	52.5 ± 3.7	49.6 ± 4.0	50.4 ± 3.4
StO_2 _difference GlycoCarn^®^	44.0 ± 3.7	46.8 ± 3.4	45.6 ± 4.6	43.5 ± 3.8	41.1 ± 3.7	41.4 ± 3.7	42.6 ± 3.7	40.4 ± 3.6	43.3 ± 3.9	42.4 ± 3.4

StO_2 _start (%) *SUPP1*	83.6 ± 1.1	90.7 ± 0.8	91.3 ± 0.7	91.6 ± 0.6	91.8 ± 0.7	92.1 ± 0.6	92.7 ± 0.5	92.5 ± 0.6	92.4 ± 0.5	92.4 ± 0.5
StO_2 _end (%) SUPP1***	38.4 ± 4.9	40.3 ± 4.6	40.7 ± 4.7	43.3 ± 4.7	42.8 ± 4.6	44.0 ± 4.4	46.2 ± 4.6	43.1 ± 4.7	43.8 ± 4.8	45.3 ± 4.9
StO_2 _difference SUPP1***	45.2 ± 4.8	50.4 ± 4.9	50.6 ± 4.7	48.4 ± 4.7	48.9 ± 4.5	48.1 ± 4.3	46.5 ± 4.6	49.4 ± 4.6	48.5 ± 4.7	47.1 ± 4.8

StO_2 _start (%) SUPP2*	85.7 ± 1.3	90.1 ± 0.9	90.6 ± 0.8	91.4 ± 0.7	91.7 ± 0.7	91.6 ± 0.7	91.9 ± 0.7	92.5 ± 0.7	91.9 ± 0.7	92.5 ± 0.7
StO_2 _end (%) *SUPP2*	38.2 ± 3.5	44.3 ± 4.1	47.2 ± 4.2	47.5 ± 3.5	50.0 ± 3.7	49.6 ± 4.3	51.1 ± 4.1	50.4 ± 4.4	51.2 ± 3.8	53.6 ± 3.7
StO_2 _difference *SUPP2*	47.5 ± 3.3	45.8 ± 3.8	43.4 ± 3.9	43.9 ± 3.4	41.7 ± 3.5	42.1 ± 4.1	40.9 ± 3.8	42.1 ± 4.0	40.8 ± 3.6	38.9 ± 3.4

StO_2 _start (%) *SUPP3*	84.2 ± 1.1	90.8 ± 0.9	91.1 ± 0.9	91.6 ± 0.8	91.7 ± 0.7	91.9 ± 0.7	92.0 ± 0.6	92.1 ± 0.6	92.4 ± 0.6	92.9 ± 0.6
StO_2 _end (%) *SUPP3*	42.9 ± 4.2	47.1 ± 4.1	47.9 ± 3.7	50.9 ± 4.0	47.9 ± 3.3	49.7 ± 3.6	49.5 ± 3.9	51.3 ± 3.9	51.0 ± 4.0	51.1 ± 4.0
StO_2 _difference *SUPP3*	41.2 ± 3.8	43.7 ± 3.9	43.2 ± 3.5	40.7 ± 3.7	43.8 ± 3.2	42.2 ± 3.4	42.6 ± 3.7	40.8 ± 3.7	41.4 ± 3.8	41.8 ± 3.7

Data are mean ± SEM.

StO_2 _Start: *Condition effect (p = 0.02); GlycoCarn^® ^higher than SUPP2. **Time effect (p < 0.0001); Set 1 lower than all other sets. No condition × set interaction (p = 1.00).

StO_2 _End: ***Condition effect (p = 0.003); SUPP1 lower than all other conditions. **Time effect (p = 0.002); Set 1 lower than sets 5-10. No condition × set interaction (p = 1.00).

StO_2 _Difference: ***Condition effect (p = 0.004); SUPP1 greater than all other conditions. No time effect (p = 0.94) or condition × set interaction (p = 1.00).

† StO_2 _values monitored continuously during the 10 set exercise protocol. Start values indicate the value prior to beginning each set of exercise. End values indicate the value at the conclusion of each set of exercise.

When removing set number from the model and only considering the condition comparison, an effect was noted for StO_2 _at the end of exercise (p = 0.003), with SUPP1 lower than all other conditions. An effect was also noted for StO_2 _difference (p = 0.003), with SUPP1 greater than all other conditions. No statistically significant difference was noted between conditions for StO_2 _at the start of exercise (p = 0.12). Data are presented in Table 5.

**Table 5:** Muscle tissue oxygen saturation data pooled over 10 sets of bench press exercise in 19 resistance trained men receiving placebo or supplement in a cross-over design.

Variable†	Baseline	Placebo	GlycoCarn^® ^	SUPP1	SUPP2	SUPP3
StO_2 _start of exercise (%)	90.9 ± 0.3	91.2 ± 0.3	91.9 ± 0.2	91.1 ± 0.3	91.0 ± 0.3	91.1 ± 0.3
StO_2 _end of exercise* (%)	47.1 ± 1.0	47.9 ± 1.3	48.6 ± 1.2	42.8 ± 1.5	48.3 ± 1.2	48.9 ± 1.4
StO_2 _difference* (start-end)	43.8 ± 1.0	43.2 ± 1.3	43.3 ± 1.2	48.3 ± 1.4	42.7 ± 1.1	42.1 ± 1.1

Data are mean ± SEM.

*Condition effect for StO_2 _end of exercise (p = 0.003); SUPP1 lower than all other conditions.

*Condition effect for StO_2 _difference (p = 0.003); SUPP1 greater than all other conditions.

No statistically significant difference noted between conditions for StO_2 _start of exercise (p = 0.12).

† StO_2 _values monitored continuously during the 10 set exercise protocol. Start values indicate the value prior to beginning each set of exercise. End values indicate the value at the conclusion of each set of exercise. The mean value of the 10 sets for each subject, under each condition, was used in data analysis.

### Muscle Pump

No statistically significant interaction (p = 0.80) or condition effect (p = 0.74) was noted for subjective muscle pump. However, a time main effect was noted (p < 0.0001), with values higher post-exercise compared to pre-exercise. No statistically significant interaction (p = 0.99), condition (p = 0.99), or time effect (p = 0.34) was noted for the circumference measure. Data are presented in Table 6.

**Table 6:** Circumference and perceived muscle pump data of 19 resistance trained men receiving placebo or supplement in a cross-over design.

Condition	Circumference (cm)	*Perceived Muscle Pump (0-10 VAS)
Baseline *Pre*	101.6 ± 1.3	1.4 ± 0.3
Baseline *Post*	102.5 ± 1.3	7.8 ± 0.2

Placebo *Pre*	101.9 ± 1.0	1.2 ± 0.1
Placebo *Post*	102.2 ± 1.1	7.5 ± 0.3

GlycoCarn^® ^*Pre*	101.3 ± 1.1	1.3 ± 0.1
GlycoCarn^® ^*Post*	102.4 ± 1.1	7.7 ± 0.3

SUPP1 *Pre*	101.3 ± 1.1	1.4 ± 0.2
SUPP1 *Post*	101.6 ± 1.1	7.9 ± 0.2

SUPP2 *Pre*	101.7 ± 1.2	1.2 ± 0.1
SUPP2 *Post*	102.2 ± 1.1	8.0 ± 0.3

SUPP3 *Pre*	101.2 ± 1.1	1.3 ± 0.1
SUPP3 *Post*	102.2 ± 1.1	7.7 ± 0.3

Data are mean ± SEM.

No statistically significant interactions or condition effects noted for either variable (p > 0.05).

* Time main effect for perceived muscle pump (p < 0.0001).

Pre = before exercise; Post = after exercise

### Biochemical Measures

No statistically significant interaction (p = 0.22) or condition effect (p = 0.20) was noted for HLa. However, a time main effect was noted (p < 0.0001), with values higher post-exercise compared to pre-exercise. No statistically significant interaction (p = 0.98), condition (p = 0.31), or time effect (p = 0.77) was noted for NOx. No statistically significant interaction (p = 0.45), condition (p = 0.33), or time effect (p = 0.19) was noted for MDA. However, MDA decreased 13.7% from pre-exercise to post-exercise with GlycoCarn^® ^and increased in placebo (9.3%), SUPP1 (37.9%), SUPP2 (1.2%), and SUPP3 (20.0%). Data are presented in Table 7.

**Table 7:** Bloodborne data of 19 resistance trained men receiving placebo or supplement in a cross-over design.

Condition	*Lactate (mmol∙L^-1^)	Nitrate/Nitrite (μmol∙L^-1^)	Malondialdehyde (μmol∙L^-1^)
Baseline *Pre*	1.85 ± 0.12	22.18 ± 2.43	0.71 ± 0.06
Baseline *Post*	5.97 ± 0.33	22.11 ± 2.43	0.76 ± 0.09

Placebo *Pre*	2.03 ± 0.22	17.74 ± 1.57	0.75 ± 0.08
Placebo *Post*	6.52 ± 0.34	19.90 ± 1.67	0.82 ± 0.10

GlycoCarn^® ^*Pre*	1.81 ± 0.13	22.72 ± 3.39	0.73 ± 0.06
GlycoCarn^® ^*Post*	6.62 ± 0.41	21.68 ± 2.39	0.63 ± 0.04

SUPP1 *Pre*	2.08 ± 0.14	23.61 ± 3.46	0.58 ± 0.07
SUPP1 *Post*	7.51 ± 0.43	23.57 ± 3.21	0.80 ± 0.11

SUPP2 *Pre*	1.89 ± 0.16	18.89 ± 2.29	0.80 ± 0.12
SUPP2 *Post*	7.20 ± 0.37	19.89 ± 2.25	0.81 ± 0.11

SUPP3 *Pre*	1.53 ± 0.12	21.92 ± 2.91	0.66 ± 0.08
SUPP3 *Post*	7.10 ± 0.31	22.33 ± 2.69	0.79 ± 0.08

Data are mean ± SEM.

No statistically significant interactions or condition effects noted for any variable (p > 0.05).

* Time main effect for lactate (p < 0.0001).

Pre = before exercise; Post = after exercise

## Discussion

Our findings indicate that, compared to a maltodextrin placebo, none of the products tested in the present study result in effects that are statistically different with regards to exercise performance, skeletal muscle blood flow, muscle pump, HLa, NOx, or MDA. These findings clearly refute the advertisement claims for these products, at least in the context of their use to impact acute exercise performance, blood flow, muscle pump, and NOx within a controlled laboratory environment. Of course, it is possible that 1) routine use of these products may result in favorable effects in our chosen variables over time (this is especially true for such ingredients as creatine and beta alanine) and/or 2) the products may influence variables that were not measured within the present design (e.g., those influencing exercise recovery; lower body exercise performance; exercise performance assessed at a higher relative intensity). Additional study would be needed to generate such data.

It is interesting to note that the single ingredient GlycoCarn^® ^(in addition to 16 grams of maltodextrin as used in the present design) results in similar or more-favorable effects in terms of blood flow (StO_2 _start of exercise; as measured by NIRS), as well as the total volume load measured during the 10 set bench press protocol. Although not of statistical significance, from a practical standpoint, the percent increases in volume load above placebo and the other supplements may be of importance to serious athletes desiring a slight performance advantage. Moreover, while a slight to moderate increase in lipid specific oxidative stress (as measured by MDA) was observed with all other conditions, the noted decrease with GlycoCarn^® ^may be of interest to those seeking antioxidant support within a pre-workout dietary supplement. Admittedly, the importance of these subtle differences in blood flow, total volume load, and MDA in relation to exercise performance and recovery are unknown at the present time and require additional study. Hence, athletes will need to consider the cost to benefit ratio when making such a decision as to whether or not to use an ingredient such as GlycoCarn^®^.

While several anecdotal reports exist indicating a performance benefit when using the products tested in the current study, we are unaware of any peer reviewed scientific manuscripts that examine any of these products. Based on the caffeine and other supposed performance aids contained within these products, we believed that it would be possible that a performance effect would be observed. However, because the actual dosage of ingredients contained within the products is unknown within a proprietary blend (see Figures 1, 2, and 3), it is possible that the actual amount of caffeine and other ingredients is simply too low to promote an ergogenic effect. In fact, studies using caffeine to improve resistance exercise performance have been mixed, as noted in a recent comprehensive review [3]. One recent study found no effect of a caffeine containing dietary supplement on resistance exercise performance, despite using a relatively high dosage of caffeine (400mg) [26]. Even this amount, which may not be adequate for many individuals, would correlate to approximately 5mg∙kg^-1 ^for subjects in the present study (based on a mean body mass of 80kg). Although not possible to determine from looking at the product labels, based on the lack of a performance effect, it is doubtful that the caffeine dosage contained within the tested products is adequate.

Aside from caffeine (and agents such as creatine and beta alanine--which need to be consumed on a regular basis in order to provide ergogenic effects), the tested products contain very few additional ingredients that have been shown in human clinical research studies to provide an ergogenic effect. Moreover, as with caffeine, the dosage of each specific ingredient may be too low to provide any benefit. Logic dictates that if a single serving has a weight of 20 grams and half of the serving is comprised of carbohydrate and flavoring, little weight remains for each of the additional 30-60 ingredients. Our data clearly show that ingredient number has no influence on product effectiveness. In fact, the use of a very inexpensive maltodextrin powder yields similar effects as all products used for comparison in this design. Considering a per-serving cost of approximately $2 (when using the amount of powder included within the present design), the reasonable choice for an athlete may simply be to use a carbohydrate powder. That being said, it should be reiterated that the tested products may provide benefit outside of the measures tested in the present design, and because of this, they may in fact be superior to maltodextrin with regards to other measures (as well as our included measures, albeit tested using a different study design). This important issue should be considered by athletes and sport nutritionists when making such a decision.

Pertaining to ingredients, the amino acid L-arginine is a component of all three supplements used in the present study, as well as most other "nitric oxide stimulating" dietary supplements sold on the market today. While L-arginine is indeed the precursor to nitric oxide biosynthesis and has been associated with enhanced vasodilatation [27,28], the rationale for inclusion of L-arginine within pre-workout supplements is primarily based on research using intravenous L-arginine, often at dosages as high as 20-30 grams, and not oral intake of L-arginine at a dosage of 3-5 grams. Studies comparing intravenous and oral L-arginine indicate no effect of oral L-arginine on vasodilatation, possibly due to variance in oral L-arginine bioavailability [29]. Additionally, studies involving oral intake of L-arginine at dosages from 10-20 grams indicate no benefit with regards to increasing nitric oxide or enhancing blood flow [30-32].

A further problem with the use of L-arginine as a nitric oxide stimulator is that L-arginine availability is likely not the rate limiting component in this reaction. Rather, nitric oxide synthase enzymes appear most important [33]. Two recent investigations provide support for this point. In one study, 3 grams per day of L-arginine was used and found not to increase nitric oxide availability, but rather reduced exercise time to fatigue in patients with peripheral arterial disease [34]. Another study involved supplementation with 6 grams per day of L-arginine in exercise trained men, and noted no effect on nitric oxide production, lactate and ammonia metabolism, or performance in intermittent anaerobic exercise [35]. Based on the above, adding L-arginine to a pre-workout powder for purposes of increasing nitric oxide is not supported by the available literature.

One final consideration is the knowledge that while brief production of nitric oxide at low (nanomolar) concentrations favor enhanced blood flow, high concentrations favor cell cycle arrest and apoptosis. Moreover, it is important to keep in mind that high levels of nitric oxide can react with superoxide anion to form peroxynitrite, a very harmful chemical [36] involved in nitrosative stress [37]. Therefore, dramatically increasing nitric oxide via use of nutritional supplements, assuming this is actually possible, does not appear desirable. Considering the findings presented here indicating a lack of increase in nitrate/nitrite, none of the tested supplements would be thought to promote an increase in peroxynitrite.

A few previous studies have used the measure of NIRS to assess tissue blood flow during resistance exercise [19-21]. Our findings are similar to those previously presented, indicating a significant decrease in StO_2 _from the start to the end of the exercise set, with a return to pre-set values within one minute of exercise recovery (data not shown). We also show here that as an exercise session continues, blood flow to the muscle is increased, as evidenced by the increase in StO_2 _at the start of exercise from set one to set two and beyond (Table 4). However, despite popular writings within fitness and bodybuilding publications indicating that nitric oxide controls skeletal muscle blood flow during exercise, scientific evidence refutes this notion, demonstrating that nitric oxide plays only a non-obligatory role in exercise hyperemia [38]. Our data support this notion, in that blood flow as measured using StO_2 _(start of exercise) increased approximately 10% from set one to set 10, despite the finding that NOx remained essentially unchanged from pre- to post-exercise (Table 7). As an aside, we believe that the inclusion of NIRS allows for the accurate measure of muscle tissue oxygen saturation, with very little error. This device may have value in future experiments designed to approximate muscle tissue blood flow with and without the use of dietary supplements.

In relation to muscle blood flow, many anecdotal reports indicate a more robust muscle pump when using pre-workout products designed to increase nitric oxide. Our data using a subjective rating scale for muscle pump, in addition to circumference measures, indicate that no such effect is observed in a controlled laboratory environment. In this regard, a placebo effect is certainly possible [39], leading individuals to believe that such an effect is absolute; as many individuals using such products are inundated with advertisements claiming increased blood flow and muscle pump. At the present time, these claims remain unsubstantiated. This phenomenon is described in detail within a recent review of nitric oxide dietary supplements for sports [2]. Admittedly, our measures of muscle pump, although performed to the best of our known abilities, are rather crude. Perhaps if a more sophisticated measure were available to assess muscle pump, we may have noted condition differences. However, even if this were the case, the main findings of no difference in performance measures may overshadow any potential effects for muscle pump.

Our findings for no change in NOx with GlycoCarn^® ^refute our initial work, in which we have noted an increase in both resting [14] and stress-induced NOx [13]. The discrepancies in findings may be due to the fact that in the present design we simply provide a single serving of GlycoCarn^® ^prior to exercise, whereas our prior work involved four [13] or eight [14] weeks of GlycoCarn^® ^treatment. Likewise, our data are in opposition to the work of Jacobs and colleagues [12] who recently reported an improvement of 2.6-15% in high intensity cycle sprint performance with 4.5 grams of GlycoCarn^® ^compared to a placebo. In this same study these investigators also noted an approximate 16% decrease in post-exercise blood HLa with GlycoCarn^® ^compared to placebo. Differences in the exercise protocol likely contributed to the discrepancy in findings between the two studies. Finally, we have noted previously that GlycoCarn^® ^results in lower resting MDA following chronic intake [14]. The present study extends those findings by noting a decrease, albeit statistically insignificant, in MDA from pre- to post-exercise, indicating a potential antioxidant effect. Interesting to note, this favorable effect of GlycoCarn^® ^on MDA reduction was associated with the highest StO_2 _at the start of exercise, indicating a possible association between increased blood flow and decreased lipid peroxidation. The converse was also true, as SUPP1 demonstrated the greatest increase in MDA from pre- to post-exercise, while displaying the lowest StO_2 _at the start of exercise and the greatest drop in StO_2 _from the start to the end of exercise. These findings support the idea that exercise-induced hypoxia is associated with increased lipid peroxidation, likely due to increased free radical production [24].

It is possible that chronic treatment of GlycoCarn^® ^may result in more robust changes in MDA or other markers of oxidative stress. Using a different stress protocol (handgrip dynamometry vs. resistance exercise), we have reported recently that four weeks of GlycoCarn^® ^treatment at a daily dosage of 4.5 grams in resistance trained men results in a 45% decrease in oxidized to total glutathione ratio [40]. Additional work is needed to determine the antioxidant effect of chronic GlycoCarn^® ^administration following resistance exercise, and to determine whether or not such an effect translates into improved post-exercise recovery.

One explanation for our lack of a performance effect for the chosen supplements, in addition to GlycoCarn^®^, could be our specific sample of subjects. That is, they may have been non-responders to treatment, as has been reported previously for a variety of sport supplements including caffeine [41], creatine [42], and GlycoCarn^®^, in terms of nitrate/nitrite [13]. If this were true, it is possible that a different group of subjects may have responded positively to treatment. This should be considered when athletes are contemplating the use of such products. For example, of our 19 subjects, 11 responded positively to GlycoCarn^® ^in terms of total volume load, with a mean improvement above placebo of 12.6%. This is in opposition to the 3.3% improvement above placebo when including all 19 subjects in the analysis. Of course, serial studies are needed to note the number of individuals who consistently and reliably respond to treatment. As with most nutritional supplements, the simple reality is that some individuals will likely respond well to treatment (i.e., experience a noted improvement in performance and/or some other variable of interest), while others will likely experience no benefit. In this case, individual experimentation is needed.

## Conclusion

We conclude that when compared to a maltodextrin placebo, none of the products tested in the present study resulted in effects that are statistically different with regards to exercise performance, skeletal muscle blood flow, muscle pump, HLa, NOx, or MDA. The single ingredient GlycoCarn^® ^(combined with 16 grams of maltodextrin) resulted in the highest StO_2 _at the start of exercise and a reduction in exercise-induced lipid peroxidation, as measured by plasma MDA. Although not of statistical significance, SUPP1 resulted in a greatest power output during the bench press throws compared to the placebo and other conditions (range: 0.4%-5.8%), and GlycoCarn^® ^resulted in a greater total volume load compared to the placebo and the supplements tested (range: 2.5%-4.6%). These data indicate that 1) a single ingredient (GlycoCarn^®^) can provide similar practical benefit as compared to finished products containing multiple ingredients pertaining to many of the outcome measures included within the present design, and 2) the tested finished products are clearly ineffective in terms of increasing blood flow and improving acute upper body exercise performance, and do not produce results that match the widely advertised marketing claims. These concluding statements should be considered within the context of the current study design, and may not be generalized to other designs inclusive of different exercise modes and intensities, and/or different outcome measures.

## Competing interests

RJB has been the Principal Investigator on research grants funded by Sigma-Tau HealthScience since 2005. He has also received research funding or acted as consultant to other nutraceutical and dietary supplement companies including Mannatech, OmniActive Health Technologies, Kaneka Nutrients, Danisco, Minami Nutrition, Jarrow Formulas, National Safety Associates, Vital Pharmaceuticals, Champion Nutrition, Experimental and Applied Sciences, Purus Labs, and CE-Bio. All other authors declare no competing interests.

## Authors' contributions

RJB was responsible for the study design, overseeing data collection, performance of biochemical assays, statistical analysis, and preparation of the manuscript. TMF, JFT, CGM, and REC were responsible for data collection/entry and assistance with manuscript preparation. BKS was responsible for the study design and assistance with manuscript preparation. All authors read and approved the final manuscript.
